# Main and interactive effects of inflammation and perceived neighbourhood cohesion on psychological distress: results from a population-based study in the UK

**DOI:** 10.1007/s11136-019-02143-7

**Published:** 2019-02-25

**Authors:** Efstathios Papachristou, Eirini Flouri, Theodora Kokosi, Marta Francesconi

**Affiliations:** Department of Psychology and Human Development, UCL Institute of Education, University College London, 25 Woburn Square, London, WC1H 0AA UK

**Keywords:** CRP, Inflammation, Neighbourhood cohesion, Psychological distress, Understanding society

## Abstract

**Purpose:**

Low neighbourhood cohesion and increased levels of inflammation are independent predictors of psychological distress. In this study we explored if they also interact to predict it.

**Methods:**

Our sample was 9,393 adult participants of the UK Household Longitudinal Study (UKHLS), a large longitudinal household panel study in the UK. Inflammation was measured using C-reactive protein levels. Perceived neighbourhood cohesion was measured using a 13-item questionnaire. Psychological distress was measured with the General Health Questionnaire-12.

**Results:**

Perceived neighbourhood cohesion and inflammation retained their significant main effects on psychological distress even after adjustment for confounders (age, gender, ethnicity, partner status, education, smoking status, obesity and urbanicity). The effect of neighbourhood cohesion was larger. However, we did not find evidence for an interactive association between the two.

**Conclusions:**

Perceived neighbourhood cohesion was inversely related to psychological distress, over and above other important person- and neighbourhood-level characteristics. Inflammation was also associated with psychological distress, albeit less strongly. If these associations are causal, they suggest that promoting neighbourhood cohesion can alleviate some of the burden associated with psychological distress.

## Introduction

Psychological distress is an established correlate of physical health problems [1–3], health behaviours such as smoking and alcohol use [4, 5], and increased all-cause mortality [6]. It is also common in the general population, with prevalence rates ranging from 3.1% for severe to 15.1% for moderate psychological distress in the U.S. for example [7]. To date, there have been numerous attempts to identify risk factors for psychological distress at the individual (person) level. Nonetheless, there is also evidence suggesting that influences at the community and neighbourhood levels may be important [8–11]. From a public health perspective, identification of such higher-level influences is particularly valuable for service and intervention planning.

Some of the neighbourhood characteristics that are consistently shown to be associated with psychological distress in the available literature include deprivation [12–16] and safety [17, 18]. Neighbourhood deprivation (usually approximated by neighbourhood poverty and low socioeconomic status) seems to be associated with psychological distress via stress. Neighbourhood deprivation is related to crime, violence and social isolation, but also adverse physical conditions such as poor quality housing and environmental toxicants [15, 19]. Chronic exposure to such stressors is known to result in accumulating “wear and tear” of the physiological systems of the body, a process termed allostatic load, due to the body’s chronic attempts to regulate optimal functioning under conditions of challenge or demand [20]. Exposure to environments eliciting allostatic processes has been shown to impact primarily on the endocrine and inflammatory response systems [21, 22], in turn implicated in psychological distress. For example, a meta-analysis of cross-sectional studies by Howren et al. [23] suggests that increased levels of inflammatory markers such as interleukin 6 (IL-6) and C-reactive protein (CRP) are significantly associated with an increased risk of depression even after adjustment for confounding factors [23]. There is now compelling evidence that there are additional direct effects of inflammatory cascades on psychopathology at the cellular level [24–26] but also via epigenetic effects on the expression of genes whose variation is linked to risk for depression and stress-related disorders [27]. Longitudinal studies in general-population samples of both young and older adults provide further support for a direct link between increased levels of inflammation and mental illness, in particular depression [28], albeit the direction of the association is unclear [29], effect sizes are generally small, and findings in some of the studies are gender specific [30].

The relationship between other neighbourhood characteristics, in particular cohesion, with psychological distress appears to be more complicated. Cross-sectional studies document a significant association between the two [12, 13, 31], yet the longitudinal effect of neighbourhood social cohesion on psychological distress or, generally, mental health is unclear [32, 33], suggesting a potentially complex causal pathway. Overall, it appears that social cohesion among neighbours may lead to a higher degree of social organisation, such as the provision of instrumental support, which, in turn, is linked to higher levels of well-being [34]. This is a plausible pathway given that social cohesion is typically approximated by common values and a civic culture, social order and social control, social solidarity, social networks and social capital, and place attachment [35]. Recent attempts to disentangle empirically the role of neighbourhood cohesion in the development of psychological distress examined the effect of its interactions with other neighbourhood-level characteristics, producing mixed results. For example, living in a socially cohesive neighbourhood was shown to modify the effect of neighbourhood deprivation [36], but not neighbourhood safety [17], on mental health. What has not yet been examined are possible interactions of neighbourhood cohesion with inflammation, which, as discussed, is linked to psychological distress and may be one of the biological mechanisms through which stressors lead to psychological distress. As the effect of exposure to stressors is in turn attenuated in supportive environments, we explored, for the first time, if a socially cohesive neighbourhood may be one such environment. This allowed us to examine whether the effect of inflammation on mental health varies by level of neighbourhood cohesion. Exploring interactive associations with inflammation to predict psychological distress has been done with individual-level variables. For example, there is a significant and positive association of the interaction between level of education and inflammation with psychological distress, indicating stronger associations of psychological well-being with inflammation among those with lower education [37].

Although the interaction between neighbourhood cohesion and inflammation has not been investigated, the extant research has explored, and showed, links between inflammation and neighbourhood deprivation and neighbourhood safety [38–42]. The link with neighbourhood cohesion however is less robust. Although people residing in neighbourhoods perceived as less cohesive display greater affective reactivity to daily stressors [43], two recent studies showed that neighbourhood cohesion was one of the weakest predictors of inflammatory and other physiological processes compared to other neighbourhood characteristics [21, 38]. In the first study, by Robinette et al. [21], neighbourhood cohesion was a significant predictor of only two of the seven regulatory systems used to calculate a summative allostatic load variable [21]. The second study, by Nazmi et al. [38], reported null findings for the association between perceived cohesion and fibrinogen and IL-6 levels [38]. Taken together, the available evidence suggests that the relationship between neighbourhood cohesion and psychological distress is not mediated by inflammation. It is, however, possible, as already discussed, that it may be moderated by it, such that neighbourhood cohesion and inflammation may interact with one another to impact on psychological distress. We expected that, in line with much health research showing multiplicative effects with neighbourhood characteristics [11], there may be increasing probability of poor mental health with decreasing neighbourhood cohesion as well as with increasing inflammation. Stated differently, we hypothesised that people living in neighbourhoods that they perceive as less cohesive and have higher inflammation would show higher levels of psychological distress compared to (a) people with low levels of inflammation who perceive their neighbourhoods as less cohesive, or (b) those who perceive their neighbourhoods to be cohesive but have higher levels of inflammation. We expected this even after accounting for individual and neighbourhood-level confounders (i.e. variables associated with psychological distress, inflammation and neighbourhood cohesion) including obesity, education, ethnicity, urbanicity and smoking but also neighbourhood safety.

We tested this hypothesis in a large general-population UK study, which, importantly [14], measured neighbourhood cohesion well. Neighbourhood cohesion in the studies to date is typically approximated by self-reported quality of relationships with neighbours [12, 21, 38] or sense of community and attraction to the neighbourhood [13], or by culturally appropriate measures such as network and neighbourhood homogeneity [44]. In our study, we measured perceptions of neighbourhood cohesion using a 13-item instrument assessing all three.

## Methods

### Sample

We used data from the UK Household Longitudinal Study (Understanding Society), an annual longitudinal survey of over 40,000 households (at wave 1) in all four UK countries. Understanding Society includes the larger general-population sample (GPS) [45], a stratified clustered random sample of households recruited in 2009–2011 (wave 1) and a smaller component of the pre-existing British Household Panel Survey (BHPS) [46]. There have been seven waves of interviews thus far. Biomedical measures including CRP, fibrinogen and body mass index (BMI) were taken during a nurse visit approximately 5 months after the mainstage interview. In wave 2 (2010–2012), the nurse health assessments were conducted with a subset of the GPS component, with data collection extending over 24 months. In wave 3 (2011–2013), they were conducted with the BHPS sample component. The time period was the same as the second year of data collection for the GPS. Participants were eligible to participate in the nurse visit if they had taken part in the corresponding main interview in English, were at age 16 years and above, lived in England, Wales or Scotland (nurse visits were not conducted in Northern Ireland) and were not pregnant [47]. Further details of the sampling and timelines associated with the data collection can be found at http://www.understandingsociety.ac.uk/documentation. Understanding society has been approved by the University of Essex ethics committee. Approval from the National Research Ethics Service was obtained for the nurse health assessment.

**Fig. 1:**
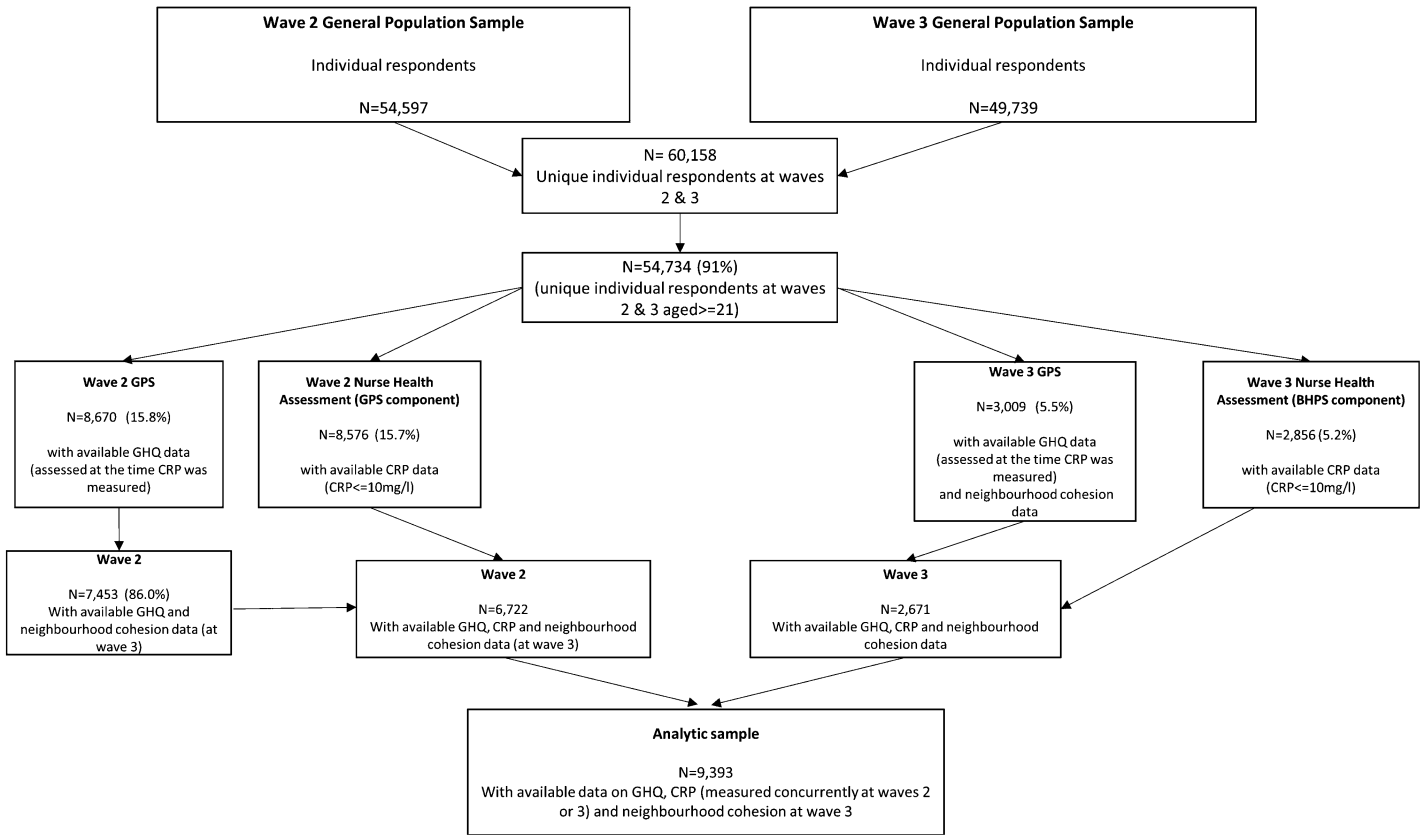
Flow chart of the study

Our study used data from both the GPS and the BHPS participants who took part in either wave 2 or 3, when CRP and fibrinogen data were collected. Information of participants from wave 1 was added to reduce the amount of missing data in the covariates. The flow chart of the study is illustrated in Fig. 1. As can be seen, we included in the analytic sample (*n* = 9,393) respondents who: (a) were at least aged 21 years (ages ranged 21–97 years); (b) had data on CRP (see further in Measures); (c) had data on the general health questionnaire (GHQ), our measure of psychological distress, at the time of the measurement of CRP and (d) had data on perceived neighbourhood cohesion and neighbourhood safety. Of the analytic sample, 4,170 (44.4%) participants were males and 5,223 (55.6%) were females.

### Measures

C-reactive protein (CRP) and fibrinogen were measured at wave 2 or 3 as part of the nurse health assessment, as explained. CRP was analysed from serum using the N latex CRP mono immunoassay on the Behring Nephelometer II analyzer (Dade Behring, Milton Keynes, UK). Intra- and inter-assay coefficients of variation were less than 2%. In line with previous research, participants with CRP levels higher than 10 mg/L (likely due to infection) were excluded. Fibrinogen was analysed from citrate plasma samples using a modification of the Clauss thrombin clotting method on the IL-ACS-TOPS analyser. Intra- and inter-assay coefficients of variation were less than 7%. We modelled CRP and fibrinogen as binary indicators whereby participants in the upper tertile of their distributions were classified as having high CRP or fibrinogen, respectively. We used an additional classification of having a low (< 1.00 mg/L), moderate (> 1.01 and < 3.00 mg/L) or high (> 3.01) level of CRP, in line with previous literature on the effects of this inflammatory marker on psychological distress [48].

Psychological distress was measured (at wave 2 or 3) with the general health questionnaire-12 (GHQ-12), a 12-item self-administered screening measure for minor psychiatric disorder [49]. The questionnaire detects changes in normal functioning and caseness (the probability that an individual has a minor psychiatric disorder). The items focus on the inability to carry out normal activities and the appearance of new and distressing symptoms. They also cover feelings of strain, depression, inability to cope, anxiety-based insomnia and lack of confidence, and are answered on a 4-point scale (1 = better than usual, 2 = same as usual, 3 = less than usual, 4 = much less than usual). We followed the scoring procedure by Goldberg and Williams [50], according to which the first two of the four response categories are scored 0 and the latter two 1, before deriving a summative score [50]. The range of the scores is therefore 0–12, with higher scores indicating higher levels of psychological distress. We converted this score into a binary variable using the generally accepted cut-off of four for caseness [51]. The reliability of the scale was excellent at both waves (Cronbach’s alpha = 0.91 and 0.90 at waves 2 and 3, respectively).

Perceptions of neighbourhood cohesion and neighbourhood safety were measured (at wave 3 only) using 13 items: (1) ‘Overall do you like living in this neighbourhood (Yes/No)?. (2) ‘I am going to read out a set of statements that could be true about your neighbourhood. Please tell me how much you agree or disagree that each statement describes your neighbourhood (a) First, this is a close-knit neighbourhood; (b) People around here are willing to help their neighbours; (c) People in this neighbourhood can be trusted; (d) People in this neighbourhood generally don’t get along with each other.’ (Response options for items 2a to 2d ranged from 1-Strongly agree to 5-Strongly disagree.) 3. ‘Here are some statements about neighbourhoods. Please enter the number that indicates how strongly you agree or disagree with each statement: (a) I feel like I belong to this neighbourhood, (b) local friends mean a lot, (c) advice is obtainable locally, (d) I can borrow things from neighbours, (e) I am willing to improve neighbourhood, (f) I plan to stay in neighbourhood, (g) I am similar to others in neighbourhood, (h) I talk regularly to neighbours. (Response options for items 3a to 3h ranged from 1-Strongly agree to 5-Strongly disagree.) 4. ‘Now I have some questions about crime. Do you ever worry about the possibility that you, or anyone else who lives with you, might be the victim of crime? Is this a big worry, a bit of a worry, or an occasional doubt?’ (yes/no). 5. ‘How safe do you feel walking alone in this area after dark? (1-Very safe to 5-SPONTANEOUS: Never goes out after dark)’. All items were recoded into binary variables in accordance with the scoring procedure followed by Emerson et al. (2014) with “0” reflecting less and “1” reflecting more neighbourhood cohesion [52]. The 13 binary items showed satisfactory internal consistency (Cronbach’s alpha = 0.80). An exploratory factor analysis showed that they loaded onto a single factor (one factor had eigenvalue > 1) suggesting that they form a valid single construct. We confirmed this finding by means of a confirmatory factor analysis (CFA) using all 13 items as categorical indicators of a single factor. Using a mean- and variance-adjusted weighted least squares (WLSMV) estimator the results of the CFA suggested good fit of the 1-factor model to the data as all fit indices were within or very close to- the recommended cut-offs [53] [comparative fit index (CFI) = 0.94; standardised root mean squared residual (SRMR) = 0.07; root mean squared error of approximation (RMSEA) = 0.05]. Therefore, they were combined into a single summative scale of perceived neighbourhood cohesion (range 0–13) on which higher scores indicate greater neighbourhood cohesion. Items 4 and 5, indexing perceived neighbourhood safety, were treated as independent exposure variables in the analyses. Of the participants in the analytic sample, 380 moved to their current address in 2009 (the beginning of the wave 1 period), 149 in 2010, 21 in 2011, 1 in 2012 and no-one moved to their current address in 2013 (the end of the wave 3 period), suggesting that scores on perceived neighbourhood cohesion and safety were not likely affected by recent changes in neighbourhood residence.

Key covariates included *age* in years, *gender, ethnicity* (white or not), *partner status* (partnered/unpartnered), *education* (university degree or not), health-related behaviours *(smoking status* and *obesity)* and *urbanicity*. Smoking status indicated whether the respondent was a current smoker or was a former smoker/never smoked. *Obesity* was defined as a BMI of 30 or higher. Finally, *urbanicity* was included in the models as a binary indicator classifying the participant’s address as being in an urban or a rural area. This information was derived from the Office for National Statistics’ rural and urban classification. According to this, urban are settlements with a population of at least 10,000.

### Statistical analysis

We began with a sensitivity analysis to examine differences in the mean values and distributions of the study variables between the analytic (*N* = 9,393) and non-analytic sample (*N* = 45,341). Continuous variables were compared using t-tests and categorical variables using chi square tests. The next step included the computation of Spearman’s correlation coefficients for the bivariate associations between the study measures. Finally, we ran a series of logistic regression models to examine the main and interaction effects of neighbourhood cohesion and inflammation on GHQ. The first model (Model A) included only gender, age and neighbourhood cohesion as predictors of the dichotomised GHQ scores. In the next model (Model B) we added CRP. In the final model (Model C) we further adjusted for our covariates: education, ethnicity, partner status, urbanicity, smoking status, obesity and perceived neighbourhood safety. We then re-ran the latter two models after including the multiplicative interaction term between neighbourhood cohesion and CRP. We ran two sensitivity analyses. For the first, we re-ran the adjusted models with and without the interaction term using fibrinogen instead of CRP as a marker of inflammation. For the second, we re-ran Models B and C using the alternative 3-level classification of participants with low, moderate and high CRP. Those with low inflammation served as the reference category. In order to quantify the risk of perceived neighbourhood cohesion on psychological distress in Model C, we computed the Cohen’s d effect size measure for their association. To do so, we standardised (z-scored) the perceived neighbourhood cohesion scale score so that the odds ratios (OR) obtained were also standardised. Next, we reversed the scores of the scale so that the exposure and outcome variables of interest measured “negative” outcomes in the same direction. After re-running the adjusted model with the transformed perceived neighbourhood cohesion score we used the formula $d = \log OR*\sqrt 3 /\pi$ to convert the newly-calculated OR into a Cohen’s d effect size measure. We calculated the CI of d using $d \pm 2 \times S{E_{\log OR}}$, where $SE_{{\log OR}} = \log \left( {CI_{{upper}} } \right) - \log \left( {CI_{{lower}} } \right)/2 \times z_{{1 - \alpha /2}}$. For the 95% CI $z_{{1 - \alpha /2}} = 1.96$.

All regression models were weighted to adjust for the unequal selection probabilities and differential nonresponse for the nurse visit. Stratification and clustering variables were also used to account for the sampling design of Understanding Society. Missing data on the covariates were handled with multiple imputation using Stata’s *mi impute* and *mi estimate* commands. The first command creates (20 in our case) imputed datasets using regression imputation and the second performs individual complete-data analyses and then uses Rubin’s combination rules to consolidate the obtained individual estimates into a single set of multiply imputed estimates. In the final model, we examined the adequacy of the imputation method using the relative increase in variance (RIV) and the relative efficiency of the predictors. RIV refers to the increase in variance due to having missing data imputed relative to the condition where no data are missing. Relative efficiency reflects the power using the number of imputations employed relative to the power that would be achieved if an unaccountably large number of imputations were used. Analyses were performed in Stata/SE 15.0 [54].

## Results

Of the 54,734 participants of Understanding Society, 9,393 were aged ≥ 21 years and had complete data on perceived neighbourhood cohesion, CRP and GHQ and therefore comprised the analytic sample. Table 1 shows the descriptive statistics of all study variables and summarises the results of a sensitivity analysis comparing the analytic and non-analytic samples. Participants included in the analytic sample were on average 5 years older and scored 0.3 points lower on the neighbourhood cohesion scale (both p-values < 0.001) than those in the non-analytic sample. They were also more likely to be female, white, rural, university-educated and a non-smoker. Finally, they were less likely to have very high CRP or GHQ scores and to report feeling unsafe in the dark. No differences were observed between the analytic and non-analytic samples with respect to obesity, partner status or worries about crime.

**Table 1:** Bias analysis of study variables between the analytic and the non-analytic samples

	Analytic sample (*n*=9,393)		Non-analytic sample (*n*=45,341)		Test
	*N*	Continuous variables			
		M(SD)	*N*	M(SD)	T
Age	9,393	53.15 (15.64)	45,341	48.45 (17.44)	− 24.18***
Perceived neighbourhood cohesion	9,393	11.54 (2.14)	27,393	11.25 (2.36)	− 10.56***

	*N*	Categorical variables			
		%	*N*	%	χ^2^
High CRP (upper tertile; >3 mg/L)	3,144	33.47	748	36.68	7.70**
High GHQ ( ≥ 4)	1,658	17.65	472	20.34	9.07**
Female	5,223	55.61	24,268	53.52	13.57***
White	6,563	96.25	26,540	78.22	1.200***
Urban	6,884	73.30	34,573	76.31	38.51***
Current smoker	1,679	30.31	8,483	39.44	156.77***
Obese	2,755	29.33	3,123	30.47	3.03
Unpartnered	3,538	39.22	203	41.26	0.81
No degree	4,386	64.17	24,643	66.55	14.57***
Feels unsafe in the dark	2,092	22.27	9,491	28.80	156.80***
Worries about crime	3,815	40.63	13,629	41.40	1.79

Means, %s and Ns are unweighted

*CRP* C-reactive protein; *GHQ* general health questionnaire

*p* < 0.05*, *p* < 0.01**, *p* < 0.001***

Of the 9,393 study participants included in the analytic sample of the study, 33% had high CRP levels, 18% had high levels of psychological distress, 96% were white, 73% lived in urban areas, 30% were current smokers, 29% were obese, 39% were unpartnered, 36% were university-educated, 22% reported feeling unsafe in the dark and 41% reported worrying about crime in their neighbourhoods. The average age of the analytic sample was 53.15 (SD = 15.64) years and the mean neighbourhood cohesion score was 11.54 (SD = 2.14).

Table 2 presents the Spearman’s correlation coefficients for the bivariate associations between the study variables. As expected, psychological distress was negatively associated with perceived neighbourhood cohesion (rho = − 0.13, *p* < 0.001) and positively associated with CRP (rho = 0.09, *p* < 0.001). Participants with high GHQ scores were also significantly more likely to be female (rho = 0.09, *p* < 0.001), to not have a degree (rho = 0.03, *p* < 0.05) or a partner (rho = 0.07, *p* < 0.001), to belong to an ethnic minority (rho = − 0.03, *p* < 0.01), to live in urban areas (rho = 0.05, *p* < 0.001), to smoke (rho = 0.11, *p* < 0.001), to be obese (rho = 0.04, *p* < 0.001) and to feel unsafe in the dark and worry about crime in their neighbourhood (rho = 0.10, *p* < 0.001 for both measures). Perceived neighbourhood cohesion and high CRP were negatively correlated (rho = − 0.03, *p* < 0.01).

**Table 2:** Spearman’s correlations for main study variables in the analytic sample (*n* = 9,393)

Variables	1	2	3	4	5	6	7	8	9	10	11	12	13
1. High GHQ	1	0.05***	− 0.13***	0.09***	0.03*	− 0.07***	− 0.03**	0.07***	0.05***	0.11***	0.04***	0.10***	0.10***
2. High CRP		1	− 0.03**	0.08***	0.08***	0.10***	0.01	0.04***	0.04***	0.06***	0.21***	0.08***	− 0.01
3. Perceived neighbourhood cohesion			1	0.01	− 0.02	0.16***	0.03*	− 0.16***	− 0.12***	− 0.12***	0.00	− 0.15***	− 0.12***
4. Female				1	− 0.02	− 0.03**	0.00	0.09***	0.00	0.04**	0.01	0.27***	0.03**
5. No degree					1	0.14***	0.08***	0.06***	0.03**	0.14***	0.11***	0.12***	− 0.02
6. Age						1	0.13***	− 0.14***	− 0.10***	− 0.24***	0.05***	0.15***	− 0.10***
7. White							1	0.01	− 0.09***	− 0.05**	0.01	− 0.02	− 0.05***
8. Unpartnered								1	0.09***	0.20***	− 0.03**	0.07***	0.00
9. Urban									1	0.07***	0.01	0.10***	0.12***
10. Current smoker										1	− 0.07***	0.02	0.02
11. Obese											1	− 0.03**	− 0.00
12. Feel unsafe in the dark												1	0.18***
13. Worries about crime													1

*p* < 0.05*, *p* < 0.01**, *p* < 0.001***

Table 3 shows the outcomes of the three regression models. In the baseline model (Model A) adjusted for age and gender, higher levels of perceived neighbourhood cohesion were negatively associated with GHQ scores ≥ 4 (OR 0.89, 95% CI 0.86–0.92, *p* < 0.001). Perceived neighbourhood cohesion retained its significant association with GHQ after adjustment for CRP (Model B) and the magnitude of the association remained unaltered (OR 0.89, 95% CI 0.86–0.92, *p* < 0.001). In this model, CRP had a significant main effect on psychological distress, with higher CRP scores being associated with scores ≥ 4 on the GHQ (OR 1.33, 95% CI 1.14–1.55, *p* < 0.001). In the third regression model (Model C) we adjusted further for covariates. As can be seen, every additional point scored on the neighbourhood cohesion scale was still associated with 8% decrease in the odds of having a score of ≥ 4 on the GHQ (OR 0.92, 95% CI 0.89–0.95, *p* < 0.001). In this model, CRP also retained its significant association with GHQ (OR 1.19, 95% CI 1.01–1.39) albeit this now became weaker and significant only at 0.05 level of significance (*p* = 0.04). With the exception of smoking, the RIV of the regression estimates of the predictors was low ranging from 0.00–0.10 while the relative efficiency of all predictors was high ranging from 0.98–100, indicating that the imputation model was adequate and the resulting estimates robust to missingness. The effect size of the association between perceived neighbourhood cohesion and psychological distress adjusted for confounders was small, albeit highly significant (Cohen’s d = 0.10, 95% CI 0.03–0.18).

**Table 3:** Logistic regression coefficients and odds ratios (95% CI) for psychological distress (GHQ-12 scores ≥ 4) in the analytic sample (*n* = 9,393)

Predictors	Model A		Model B		Model C	
	Coeff. (SE)	OR [95% CI]	Coeff. (SE)	OR [95% CI]	Coeff. (SE)	OR [95% CI]
Constant	− 0.724** (0.236)	0.485 [0.305–0.771]	− 0.756** (0.235)	0.470 [0.296–0.745]	− 1.523*** (0.317)	0.218 [0.117–0.406]
Perceived neighbourhood cohesion	− 0.118*** (0.016)	0.888 [0.860–0.917]	− 0.116*** (0.016)	0.890 [0.863–0.919]	− 0.086*** (0.017)	0.918 [0.888–0.949]
Age	− 0.004 (0.002)	0.996 [0.991–1.000]	− 0.005* (0.002)	0.995 [0.990–0.999]	− 0.003 (0.003)	0.997 [0.992–1.002]
Female	0.473*** (0.074)	1.606 [1.388–1.857]	0.446*** (0.075)	1.562 [1.348–1.810]	0.356*** (0.080)	1.428 [1.221–1.670]
High CRP			0.284*** (0.078)	1.328 [1.138–1.549]	0.171* (0.080)	1.187 [1.012–1.391]
No degree					0.105 (0.083)	1.111 [0.943–1.308]
Unpartnered					0.168* (0.075)	1.184 [1.021–1.371]
Urban					0.031 (0.089)	1.031 [0.867–1.229]
Current smoker					0.455*** (0.102)	1.577 [1.287–1.931]
White					− 0.194 (0.169)	0.824 [0.591–1.148]
Obese					0.247** (0.083)	1.281 [1.088–1.507]
Feels unsafe in the dark					0.305** (0.091)	1.356 [1.135–1.620]
Worries about crime					0.372*** (0.081)	1.450 [1.237, 1.670]

*p* < 0.05*, *p* < 0.01**, *p* < 0.001***

*CRP* C-reactive protein

Next, we added the multiplicative interaction term between perceived neighbourhood cohesion and CRP to regression Models B and C to test whether inflammation and perceived neighbourhood cohesion interact to predict psychological distress. In both models, the interaction term did not reach statistical significance levels (*p* = 0.19 and *p* = 0.17, respectively), suggesting absence of an interactive relationship.^1^

### Sensitivity analyses

We further examined the relationship between perceived neighbourhood cohesion with psychological distress adjusted for covariates (Model C) using fibrinogen instead of CRP as a marker of inflammation.^2^ The results suggested that, even when adjusting for fibrinogen, neighbourhood cohesion retained its significant association with psychological distress (OR 0.91, 95% CI 0.88–0.95, *p* < 0.001), however fibrinogen did not have a significant direct effect on GHQ (OR 0.98, 95% CI 0.81–1.18, *p* = 0.84). We then tested whether the interaction term between fibrinogen and perceived neighbourhood cohesion was significantly associated with psychological distress, however the results showed that it was not (OR 0.95, 95% CI 0.88–1.02, *p* = 0.13).

We also tested whether the results in Models B and C held using the alternative 3-level classification of inflammation level (low, moderate, high). As expected given the small effect size of inflammation on psychological distress, only CRP > 3.01 mg/L (high) (OR 1.44, 95% CI 1.19–1.73, *p* < 0.001) but not CRP between 1.01 and 3.00 mg/L (moderate) (OR 1.16, 95% CI 0.97–1.38, *p* = 0.11) was associated with increased psychological distress compared to CRP < 1.00 mg/L (low). The same pattern of results was observed after adjustments for the covariates in Model C (OR 1.22, 95% CI 1.00–1.48, *p* = 0.05; and OR 1.07, 95% CI 0.89–1.29, *p* = 0.46, respectively). The regression coefficients of neighbourhood cohesion in these models remained substantively identical to the ones obtained in the original Models B and C using the dichotomous CRP indicator. In addition, as in the original models, the interaction between neighbourhood cohesion and CRP did not reach statistical significance in either the unadjusted (*p* = 0.35) or the adjusted model (*p* = 0.33).

## Discussion

We carried out this study to explore the main and interactive effects of perceived neighbourhood cohesion and inflammation on adult psychological distress in a large general-population study in the UK. In line with previous research, we found that neighbourhood cohesion was negatively associated with psychological distress [12, 13, 44]. Inflammation was also related to psychological distress, although less strongly. However, the two did not interact to predict psychological distress, contrary to our expectations. It may, of course, be that perceived neighbourhood cohesion interacts with stress without directly affecting levels of inflammation. Future studies could test this as well as investigate directly how support from the broader community (approximated here by perceived neighbourhood cohesion) compares with that from family and close friends [55] in buffering stress. Such studies would be particularly relevant in light of evidence suggesting that the buffering effect of perceived neighbourhood cohesion on the impact of daily stressors on negative affect is very strong and survives adjustments for other forms of social support [43].

Although our study did not find evidence for a multiplicative interaction effect between perceived neighbourhood cohesion and inflammation, it showed that perceived neighbourhood cohesion had a robust effect on psychological distress. That is, even after adjustment for perceived neighbourhood safety and other confounders known to be associated with psychological distress and neighbourhood cohesion, such as obesity, smoking and urbanicity [12, 56–59], people who felt that they lived in more socially cohesive neighbourhoods had better mental health. In fact, perceived neighbourhood cohesion had the largest effect of all the variables included in our models on the risk of clinically significant psychological distress. From a public health perspective this is a finding of particular importance as it suggests that, if these associations are causal, promoting social cohesion in neighbourhoods could alleviate psychological distress and therefore the burden associated with it.

To the best of our knowledge, this is the first study to examine potential interactions between inflammation, perceived neighbourhood cohesion and psychological distress. Its additional strengths are the large sample size and the wide age range, covering the entire adult lifespan. The data come from the UK’s largest household longitudinal study, additionally characterised by low attrition rates [60]. Another advantage is the use of a broad definition of neighbourhood cohesion, covering various aspects of neighbourhood connectedness and interrelatedness. Arguably, the definition of neighbourhood cohesion is an important source of variability in the results of the extant studies on its relationship with psychological distress [12, 14].

Nonetheless, four important limitations should be considered when interpreting these results. First, approximately a fifth of the total adult sample size in our dataset had available data on the main outcome measures and therefore our analytic sample ended up being selective. Second, this study is cross-sectional and as a result we cannot comment on possible long-term impacts, or the importance of extended periods of inflammation or prolonged exposures to socially disorganised communities. For the same reason, we cannot determine if the psychologically distressed find their neighbourhoods less cohesive, if perceptions of low neighbourhood cohesion contribute to psychological distress, or if the two are related simply because they share causes. Similarly, we cannot conclude that inflammation is prospectively associated with psychological distress. In fact, there is evidence suggesting the opposite direction [61]. Finally, CRP was assessed at either wave 2 or 3 of the nurse health assessment while perceived neighbourhood cohesion at wave 3 only. Although emerging evidence suggests that trajectories of inflammation are relatively stable over longer periods of time [62], it is likely—particularly owing to the small effect size of inflammation on psychological distress—that even minor fluctuations in CRP between the two assessments could have an impact on our results. Future studies should be designed taking this potential source of bias into consideration, and the results of this study should be interpreted with this caveat in mind.

Overall, our study demonstrated that perceived neighbourhood cohesion was inversely related to psychological distress, over and above other important person- and neighbourhood-level characteristics. Inflammation was also associated with psychological distress, albeit less strongly. Perceived neighbourhood cohesion and inflammation did not interact to further increase the risk of psychological distress suggesting distinct causal mechanisms, which have yet to be identified.

## Footnotes

1 We also examined whether perceived neighbourhood cohesion and CRP interact not only between them but also with urbanicity to impact on GHQ. A fully adjusted regression model including the terms for the three 2-way interactions and the 3-way interaction between perceived neighbourhood cohesion, CRP and urbanicity showed that none of the interactions was statistically significant (*p*-values ranged from 0.13–0.99). Only perceived neighbourhood cohesion (OR 0.87, 95% CI 0.79–0.96, *p* = 0.004), but not CRP (OR 1.84, 95% CI 0.37–9.16, *p* = 0.46), retained a significant main effect on GHQ.

2 In the analytic sample fibrinogen correlated significantly with CRP (rho = 0.35, *p* < 0.01) but not GHQ (rho = 0.01, *p* > 0.05).
