# Correction to: Main and interactive effects of inflammation and perceived neighbourhood cohesion on psychological distress: results from a population-based study in the UK

**DOI:** 10.1007/s11136-019-02178-w

**Published:** 2019-04-16

**Authors:** Efstathios Papachristou, Eirini Flouri, Theodora Kokosi, Marta Francesconi

**Affiliations:** Department of Psychology and Human Development, UCL Institute of Education, University College London, 25 Woburn Square, London, WC1H 0AA UK

## Correction to: Quality of Life Research 10.1007/s11136-019-02143-7

The article “Main and interactive effects of inflammation and perceived neighbourhood cohesion on psychological distress: results from a population-based study in the UK”, written by “Efstathios Papachristou, Eirini Flouri, Theodora Kokosi and Marta Francesconi”, was originally published electronically on the publisher’s Internet portal (currently SpringerLink) on 25 February 2019 without open access.

With the author(s)’ decision to opt for Open Choice the copyright of the article changed on 16 April 2019 to © The Author(s) 2019 and the article is forthwith distributed under the terms of the Creative Commons Attribution 4.0 International License (http://creativecommons.org/licenses/by/4.0/), which permits use, duplication, adaptation, distribution and reproduction in any medium or format, as long as you give appropriate credit to the original author(s) and the source, provide a link to the Creative Commons license and indicate if changes were made.

The original article has been corrected.

